# Intestinal Microbial Dysbiosis and Colonic Epithelial Cell Hyperproliferation by Dietary α-Mangostin is Independent of Mouse Strain

**DOI:** 10.3390/nu7020764

**Published:** 2015-01-22

**Authors:** Fabiola Gutierrez-Orozco, Jennifer M. Thomas-Ahner, Jeffrey D. Galley, Michael T. Bailey, Steven K. Clinton, Gregory B. Lesinski, Mark L. Failla

**Affiliations:** 1 Human Nutrition Program, Department of Human Sciences, The Ohio State University, Columbus, OH 43210, USA; E-Mails: Gutierrez-Orozco.1@osu.edu (F.G.-O.); Steven.Clinton@osumc.edu (S.K.C.); Gregory.Lesinski@osumc.edu (G.B.L.); 2 Food Innovation Center, The Ohio State University, Columbus, OH 43210, USA; E-Mails: Jennifer.Thomas-Ahner@osumc.edu (J.M.T.-A.); Galley.2@osu.edu (J.D.G.); Michael.Bailey@osumc.edu (M.T.B.); 3 Comprehensive Cancer Center, The Ohio State University, Columbus, OH 43210, USA; 4 Division of Oral Biology, College of Dentistry, The Ohio State University, Columbus, OH 43210, USA; 5 Department of Internal Medicine, The Ohio State University, Columbus, OH 43210, USA

**Keywords:** mangosteen, alpha-mangostin, inflammation, gut microbiota, inflammatory bowel diseases, ulcerative colitis, colon

## Abstract

Beverages and supplements prepared from mangosteen fruit are claimed to support gut health and immunity, despite the absence of supporting evidence from clinical trials. We recently reported that α-mangostin (α-MG), the most abundant xanthone in mangosteen fruit, altered the intestinal microbiome, promoted dysbiosis, and exacerbated colitis in C57BL/6J mice. The objective of this study was to determine whether induction of dysbiosis by dietary α-MG is limited to the C57BL/6J strain or represents a more generic response to chronic intake of the xanthone on the gut microbiota of mice. C3H, Balb/c, Nude FoxN1^nu^, and C57BL/6J mice, each demonstrating unique microbiomes, were fed standard diet or diet containing 0.1% α-MG for four weeks. Dietary α-MG significantly altered the cecal and colonic microbiota in all four strains of mice, promoting a reduction in generally assumed beneficial bacterial groups while increasing the abundance of pathogenic bacteria. Consumption of α-MG was associated with reduced abundance of Firmicutes and increased abundance of Proteobacteria. The abundance of *Lachnospiraceae*, *Ruminococcaceae*, and *Lactobacillaceae* was reduced in α-MG-fed mice, while that of *Enterobacteriaceae* and *Enterococcaceae* was increased. Dietary α-MG also was associated with increased proliferation of colonic epithelial cells, infiltration of immune cells, infiltration of immune cells and increased fluid content in stool. These results suggest that ingestion of pharmacologic doses of xanthones in mangosteen-containing supplements may adversely alter the gut microbiota and should be used with caution.

## 1. Introduction

The gastrointestinal (GI) tract is inhabited by a collection of bacteria, viruses, and single-cell eukaryotes generically referred to as the gut microbiota. These microbes play an essential role in the development of immunity both systemically and in the intestinal mucosa, as well as in the functional and structural maturation of the GI tract. In addition, the microbiota provides a protective barrier against pathogens and has a critical role in the metabolism of dietary nutrients and xenobiotics [1]. An imbalance between putative ‘protective’* versus* ‘harmful’ intestinal bacteria, also known as dysbiosis, is often associated with diseased states [2,3,4,5,6]. For instance, the microbiota of individuals with inflammatory bowel diseases (IBD) shifts to a reduced abundance of Firmicutes and Bacteroidetes and increased abundance of Proteobacteria [7,8,9]. Altered bacterial profiles are also present in the gut of obese humans and mice [2,4,10], as well as in individuals with colorectal tumors [5] and type 2 diabetes [6]. Whether such alterations in the gut microbiota are the cause or the result of the disease processes remains to be elucidated.

Dietary components affect the composition of the gut microbiota [11,12]. For example, switching from a low fat/high fiber dietary pattern to a ‘Western’ diet rich in fat and added sugar alters the composition of the microbiome [11]. Similarly, changes in the microbiota are reported for individuals consuming an animal-based diet* versus* a plant-based diet [12]. Ingestion of poorly digested carbohydrates, also known as prebiotics, has been associated with increased abundance of protective bifidobacteria in humans [13]. Like prebiotics, many plant polyphenols have limited absorption in the small intestine and are potential substrates for bacterial metabolism that may exert either adverse or beneficial health effects, either locally or systemically if absorbed [14]. 

Dietary patterns rich in fruits and vegetables have been associated with a healthy gut. This effect has been hypothesized to be due in part to polyphenols, which possess a multitude of biological effects, such as modulation of oxidative stress and anti-inflammatory and anti-carcinogenic activities [15]. Although many polyphenols are poorly absorbed, their concentrations in the lumen of the GI tract can be several hundred micromolar [16]. Because polyphenols also exert modulatory effects on the growth of bacteria, fungi, and viruses [17,18], they may also have an impact on the composition of the gut microbiota. 

The pericarp of mangosteen (*Garcinia mangostana*), a fruit native to Southeast Asia, has been used in traditional medicine as an anti-inflammatory and wound-disinfecting agent for centuries [19]. Xanthones, the most abundant polyphenolic compounds present in mangosteen pericarp, have been associated with these and other proposed health-promoting properties [20]. This has provided the basis for extensive marketing of mangosteen-containing supplements and beverages as beneficial for gut health and immune function despite modest preclinical evidence and a lack of supporting evidence from randomized trials in humans. The anti-inflammatory activity of mangosteen xanthones has been addressed by various researchers [21]. Counter to our hypothesis, we found that dietary α-MG actually exacerbated colonic inflammation and injury during chemically-induced colitis in mice [22]. Because of the relatively poor bioavailability of xanthones in humans [23], the GI tract is exposed to relatively high concentrations these compounds [24]. This directed our attention to the relatively non-selective inhibitory activities of xanthones against bacteria residing in the large intestine [20] to determine if orally consumed α-MG might affect the gut microbial community. Indeed, dietary α-MG (0.1% α-MG for four weeks) promoted dysbiosis in the colon and cecum of otherwise healthy C57BL/6 mice [22]. This alteration was associated with loose stools, increased proliferation of colonic epithelial cells, and increased infiltration of immune cells in the colonic lamina propria. These results suggested that α-MG-induced dysbiosis may have contributed to the exacerbation of inflammation during experimental colitis. Because host genotype is one of many factors affecting the host inflammatory response as well as the composition of the gut microbiota, the primary objective of the present study was to determine whether the induction of dysbiosis by dietary α-MG is impacted by mouse genetics or represents a more general response to the xanthone. Female C3H, Balb/c, Nude FoxN1^nu^, and C57BL/6J mice were fed semi-purified diet containing 0.1% α-MG for 4 weeks. Tissue-associated bacteria in the cecum and colon, as well as the histological profiles of the colon of these mice, were analyzed.

## 2. Materials and Methods 

### 2.1. Mice 

The gut microbiota of humans and mice are considered to share broad similarities [10,25]. Induction of dysbiosis by dietary α-MG in female C57BL/6 mice was recently reported by our group [22]. Thus, we have used 3 additional strains of mice to investigate the effect of α-MG on the gut microbiota. 10-week-old female C57BL/6 (Jackson Laboratories, Bar Harbor, ME), Balb/c (Jackson Laboratories), and C3H (Charles River, Wilmington, MA, USA) mice, as well as 8–9 week old athymic FoxN1^nu^ (Harlan, Indianapolis, IN, USA) were housed in the animal facilities at The Ohio State University (OSU) under sterile conditions with controlled temperature at 23 °C and a 12-h light/dark cycle. Female mice were used as in our previous study since their response to dextran sulfate sodium-induced inflammation is less severe than males [26]. Mice were acclimatized for 1 week before initiating the study and had free access to water and standard AIN93G diet.

### 2.2. Ethics Statement

All procedures were approved under Protocol #2011A00000006 and followed the guidelines by the Institutional Animal Care and Use Committee (IACUC) at The Ohio State University. 

### 2.3. Diet 

α-MG was >98% pure as assessed by NMR spectroscopy and ESIMS [27,28]. Gamma-irradiated AIN93G diet (control) and AIN93G diet containing 900 mg/kg α-MG and FDA approved dyes E102 and E133 for green color were prepared by Research Diets (New Brunswick, NJ, USA). This dose of α-MG was selected in light of recent reports indicating its safety and efficacy in reducing tumor mass in xenograft models of colon and prostate cancers [24,29]. For a mouse weighing 20 g and ingesting 2.5 g diet/day, this dose is equivalent to 112 mg/kg body weight. Thus, the equivalent dose ((112 mg/kg)/(3/37) = 9.12 mg/kg) for an adult weighing 60 kg equates to 547 mg/day [30]. This dose is achievable for individuals ingesting either 1–2 of pure α-MG dietary supplement, approximately 7 capsules of a commercially available mangosteen dietary supplement containing 80 mg α-MG/capsule [29], or 8 ounces (224 mL) of 100% mangosteen juice [23]. 

### 2.4. Experimental Groups and Tissue Collection

After the one-week acclimation to the animal facility, mice of each strain were randomly assigned to be fed either the standard AIN93G diet (control group, *n =* 5) or the AIN93G diet containing α-MG (α-MG group, *n =* 5). Mice had *ad libitum* access to tap water and their respective diets for 4 weeks. Body weight (BW) was recorded throughout the experiment. Fresh stool was collected within a time frame of 10 min of expulsion and placed in sealed microtubes to prevent evaporation. Fresh weight was recorded soon after and stool was transferred to dishes and placed in an oven at 60 °C for 24 h to determine water content. At necropsy, the colon and cecum were excised aseptically, opened and gently rinsed in sterile cold PBS. The cecum and the distal sections of the colon were transferred to sterile tubes, frozen in liquid nitrogen and stored at −80 °C until analysis of microbiota by pyrosequencing (Section 2.6.). In addition, the mid and proximal sections of the colon were collected and fixed in 10% neutral buffered formalin for 24 h and processed for histologic evaluation and immunohistochemistry analysis. 

### 2.5. Histology and Immunohistochemistry (IHC) 

For IHC analysis, 5-μm-thick, paraffin-embedded sections of the mid colon from 5 mice per group were used. T cells and macrophages were identified using CD3 and F4/80, respectively, as markers. CD3 immunostain was performed using a rabbit anti-human polyclonal antibody diluted 1:200 (Dako #A0452, Dako, Carpinteria, CA, USA). F4/80 detection was performed using a rat anti-mouse monoclonal antibody diluted 1:100 (Serotec #MCA497G, Bio-Rad, Raleigh, NC, USA). Detection and quantification were performed as previously described [22]. To evaluate epithelial cell proliferation, tissue sections were immunostained for Ki67 using a commercially available rat anti-mouse monoclonal antibody diluted 1:50 (clone TEC-3; #M7249; Dako, Carpinteria, CA, USA). Detection and quantification followed protocols previously described [22].

### 2.6. Bacterial Analyses 

Bacterial analyses were performed using bacterial tag-encoded FLX amplicon pyrosequencing (bTEFAP) at the Research and Testing Laboratory (Lubbock, TX, USA). The 16S rRNA gene sequence was amplified for pyrosequencing using a forward and reverse fusion primer. The forward primer was constructed with (5′–3′) the Roche A linker (CCATCTCATCCCTGCGTGTCTCCGACTCAG), an 8–10 bp barcode, and the forward specific universal primer 28F (5′-GAGTTTGATCNTGGCTCAG-3′). The reverse fusion primer was constructed with (5′–3′) a biotin molecule, the Roche B linker (CCTATCCCCTGTGTGCCTTGGCAGTCTCAG), and the reverse specific primer 519R (5′-GTNTTACNGCGGCKGCTG-3′). Amplifications were performed in 25 μL reactions with Qiagen HotStar Taq master mix (Qiagen Inc., Valencia, CA, USA), 1 μL of each 5 μM primer, and 1 μL of template. Reactions were performed on ABI Veriti thermocyclers (Applied Biosytems, Carlsbad, CA, USA) using the following thermal treatment: 95 °C for 5 min, 35 cycles of 94 °C for 30 s; 54 °C for 40 s; 72 °C for 1 min, followed by one cycle of 72 °C for 10 min and 4 °C hold. Amplification products were visualized with eGels (Life Technologies, Grand Island, NY, USA). Products were then pooled equimolar and each pool was cleaned with Diffinity RapidTip (Diffinity Genomics, West Henrietta, NY, USA), and size selected using Agencourt AMPure XP (BeckmanCoulter, Indianapolis, IN, USA) following Roche 454 protocols (454 Life Sciences, Branford, CT, USA). Size selected pools were then quantified and 150 ng of DNA were hybridized to Dynabeads M-270 (Life Technologies, Grand Island NY, USA) to create single stranded DNA following Roche 454 protocols (454 Life Sciences, Grand Island, NY, USA). Single stranded DNA was diluted and used in emPCR reactions, which were performed and subsequently enriched. Sequencing was done following established manufacturer protocols (454 Life Sciences). Fasta and qual files obtained from pyrosequencing were uploaded into Quantitative Insights Into Microbial Ecology (QIIME) software version 1.7.0 [31]. Clustering of sequencing reads into operational taxonomic units (OTUs) was achieved at 97% identity. OTU picking was performed in the QIIME pipeline using the Uclust algorithm [32]. Taxonomic assignment was achieved using the Ribosomal Database Project (RDP) classifier [33], employing the GreenGenes database [34]. The minimum and maximum sequence lengths were 200 and 1000, respectively. The minimum qual score was 25. The maximum number of ambiguous bases was 6 and no primer mismatch was allowed. 86.1% of sequences were retained after quality trimming. Sequences PyNAST [35] aligned by QIIME were used the Shannon index [36] as a measurement of α-diversity using cutoff depths of 1370 and 1452, 1700 and 1912, 1774 and 1646, and 1112 and 902 sequences for cecum and colon samples from Balb/c, C3H, C57BL/6, and Athymic FoxN1^nu ^strains, respectively. Unifrac analysis [37] followed by principal coordinate analysis (PCoA, based on unweighted unifrac distance) were done to characterize the diversity in the bacterial populations by using 1639 and 1574, 1893 and 2074, 1858 and 1769, and 1140 and 1113 sequences for cecum and colon, from Balb/c, C3H, C57BL/6, and Athymic FoxN1^nu ^strains, respectively, and jackknifed data (1229 and 1180, 1419 and 1555, 1393 and 1326, and 855 and 834 sequences for cecum and colon, respectively) from Balb/c, C3H, C57BL/6, and Athymic FoxN1^nu ^strains.

### 2.7. Statistical Analysis 

All data are expressed as mean ± SD. For parametric data, statistical differences were determined by one-way analysis of variances followed by Tukey’s test. Nonparametric data were analyzed by Kruskal-Wallis test followed by selected mean comparisons with Bonferroni correction. Differences were considered statistically significant at *p* < 0.05. Analyses were performed using SPSS v. 20 (IBM, Armonk, NY, USA). For pyrosequencing data, statistically significant differences in unweighted Unifrac distance were investigated by Analysis of Similarities (ANOSIM), provided by R’s vegan package version 2.0–3 [38,39] implemented into QIIME. 

### 2.8. Availability of Supporting Data 

The sequences supporting the results of this article are available in the NCBI Sequence Read Archive under the study accession number SRP040305.

## 3. Results 

### 3.1. Dietary α-MG Increases Fluid Content in Stool

**Figure 1:**
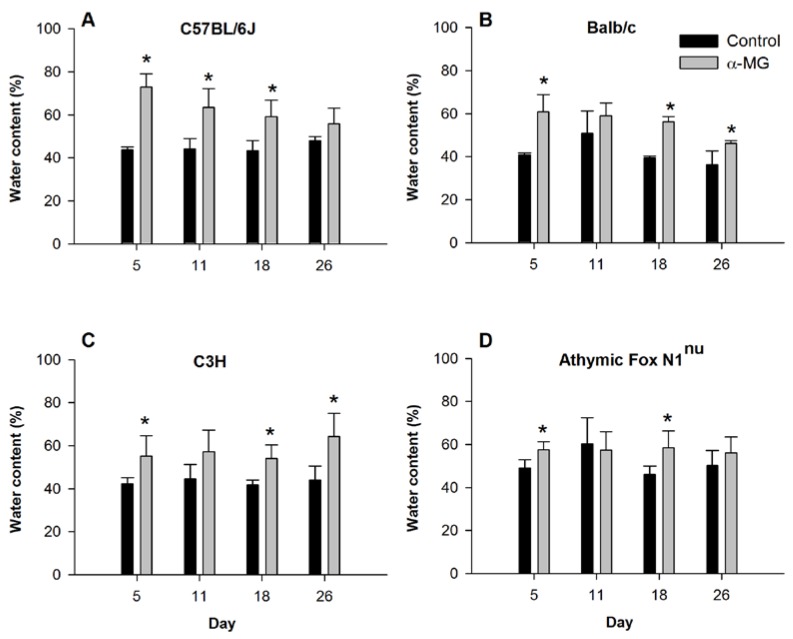
Dietary α-mangostin increases fluid content in stool. Water content in stool excreted by (**A**) C57BL/6J; (**B**) Balb/c; (**C**) C3H; and (**D**) Athymic FoxN1^nu^ mice during feeding of control diet or diet with α-MG. Stool was collected on the indicated days after initiating dietary intervention. * *p* < 0.05 against control group. Data represent the mean (±SD) of values for 3–5 mice per group for each murine strain on indicated days of the study.

Consumption of diet containing α-MG did not result in significant differences in either food intake or body weight compared to ingestion of control diet for any of the four murine strains (Table 1). However, consumption of α-MG resulted in a rapid and significant increase in the fluid content of stools for all four strains (Figure 1). Fluid content of stool was 29%, 20%, 13%, and 8.5% greater in mice fed α-MG at five days after initiating the dietary intervention in C57BL/6J, Balb/c, C3H and athymic FoxN1^nu^ strains, respectively, compared to control mice. Although this difference in stool fluid content between the dietary treatment groups tended to decline for the C57BL/6J and Balb/c mice as the weeks progressed, fluid content of stool from Balb/c and C3H mice fed diet with α-MG for 26 days remained significantly elevated compared to their respective controls fed the diet without α-MG. Athymic FoxN1^nu^ also had significantly greater water content in stool when fed α-MG for 18 days, although there was no difference between control and α-MG groups after 26 days.

**Table 1:** Body weight (g) and average food intake (g/mouse/day) of mice fed control diet or diet with α-MG for 4 weeks.

Strain	Group	Body Weight (g)/Experimental Day										Food Intake (g/Day)	
		1		6		12		19		26			
		Mean	SD	Mean	SD	Mean	SD	Mean	SD	Mean	SD	Mean	SD
**C57BL/6J**	control	18.4	0.6	19.2	1.1	19.5	1.1	21.0	1.5	20.2	0.6	2.1	0.2
	α-MG	18.7	1.9	19.0	1.6	19.4	1.3	19.8	1.3	20.2	1.1	2.2	0.3
**Balb/c**	control	19.9	1.1	20.6	1.0	20.5	1.1	20.4	1.0	21.8	1.4	2.3	0.4
	α-MG	20.5	1.0	20.6	0.6	21.4	0.8	21.6	0.8	21.7	0.3	2.3	0.2
**C3H**	control	25.2	1.2	25.0	1.4	25.3	0.8	26.2	1.1	26.8	1.7	2.7	0.2
	α-MG	25.6	1.1	25.4	1.6	25.7	2.0	26.5	2.3	27.2	1.9	2.8	0.3
**Athymic**	control	22.1	1.4	22.4	1.5	23.7	1.3	24.2	1.6	23.5	1.0	3.0	0.1
**FoxN1^nu^**	α-MG	23.0	0.9	23.6	1.0	24.2	0.8	24.8	1.2	25.0	1.4	2.9	0.1

Data are means ± standard deviation for *n =* 5 mice/group; *p* > 0.05 for body weight and food intake between dietary groups within each strain.

### 3.2. Colonic Epithelial Cell Proliferation and Immune Cell Infiltration are Stimulated by α-MG 

**Figure 2:**
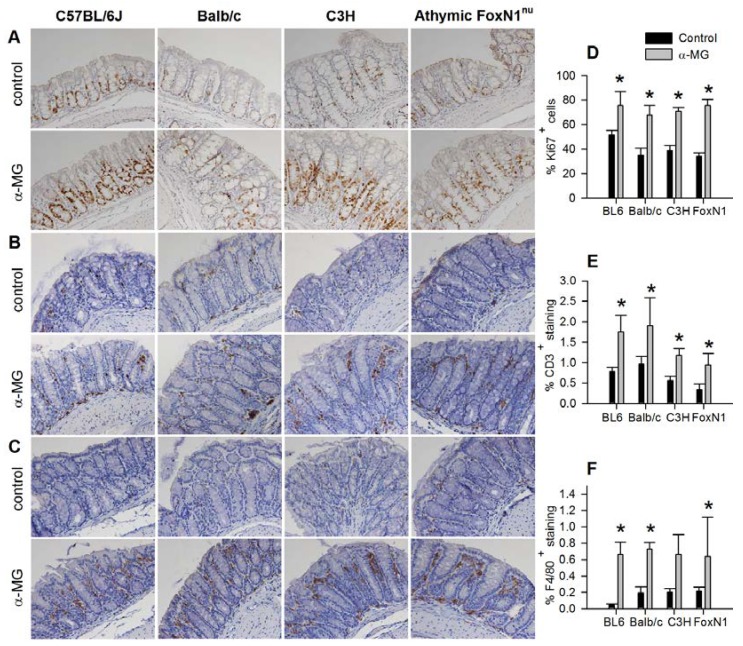
Induction of colonic epithelium hyperproliferation and infiltration of immune cells by dietary α-mangostin. Dietary α-mangostin induces a significant increase in Ki67^+^ (**A**,** D**); CD3^+^ (**B**,** E**); and F4/80^+^ (**C**,** F**) immunostaining in the mid colon of C57/BL6J, Balb/c, C3H and Athymic FoxN1^nu^ mice. * *p <* 0.05 against control group. Data are mean (±SD) for 5 mice per group for each strain fed either control diet or diet containing α-MG.

To assess epithelial cell proliferation, Ki67^+^ cells were quantified in the mid colon of C3H, Balb/c, and athymic FoxN1^nu^, as well as in C57BL/6J mice to verify our previous observation with this strain [22]. There was a significant increase in Ki67^+^ cells in the mid colonic epithelium of all four strains of mice fed diet with α-MG compared to control animals (Figure 2A). Infiltration of CD3^+^ and F4/80^+^ cells, markers of T cells and macrophages, respectively, in the mid colon also was significantly increased in all four strains of mice fed diet with α-MG compared to control diet (Figure 2B,C).

### 3.3. Dietary α-MG Induces Dysbiosis in Mice with Different Genetic Backgrounds

Because host genetics are known to impact immune function and the composition of the gut microbiota [40], we sought to determine if diet with α-MG influenced the gut microbiota in a strain-specific or strain-independent manner. The tissue-associated bacterial communities in the colon and cecum of the four murine strains were determined after feeding either control diet or a diet with α-MG for 4 weeks. Rather than using individual primers as for our previous report with the C57BL/6J mice [22], a universal bacterial primer was used for the pyrosequencing analysis in the present study. Thus, samples from our previous study using the C57BL/6J strain were re-analyzed to verify our earlier observations.

#### 3.3.1. C57BL/6J Strain

**Figure 3:**
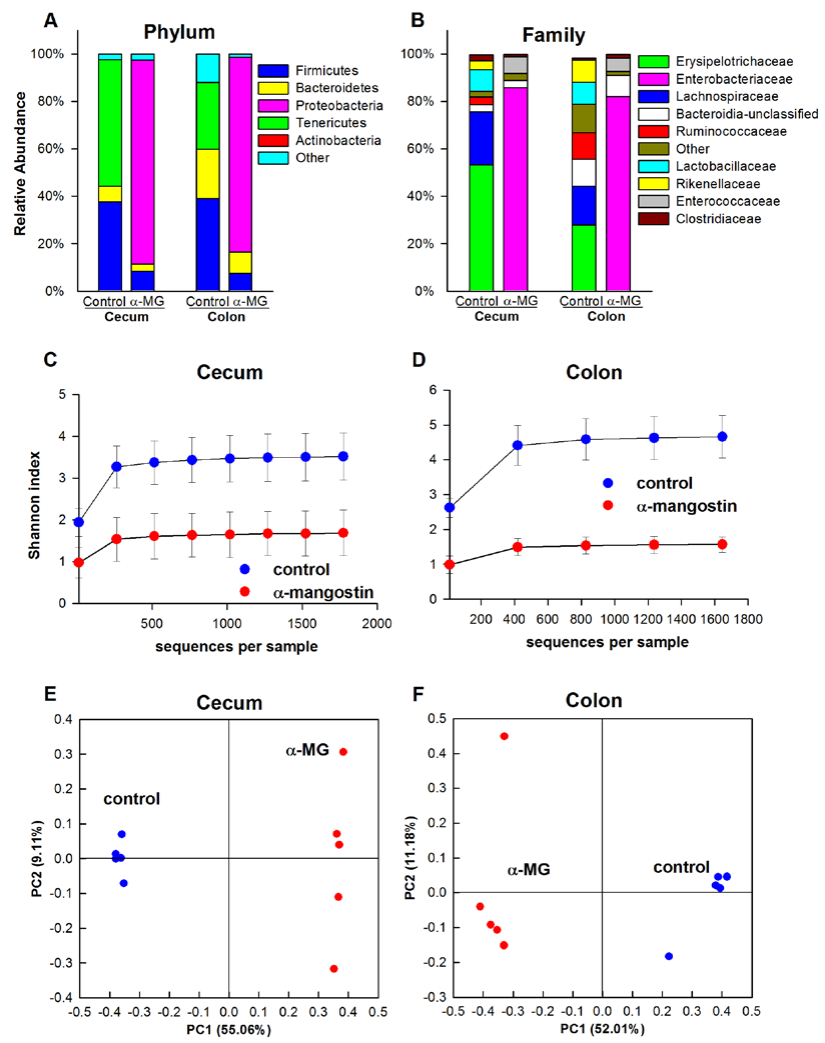
Induction of dysbiosis in C57BL/6J mice fed a diet containing α-MG. Dietary α-mangostin (α-MG) changes the relative abundance of bacterial communities in the cecum and colon of C57BL/6J mice at the (**A**) phylum and (**B**) family levels. Mice fed α-MG had significantly lower α-diversity in (**C**) cecum and (**D**) colon compared to mice fed control diet. Panels E and F show principal coordinate analysis (PCoA) for cecum and colon, respectively. Samples in the α-MG group (red) cluster together and away from those in control group (blue). Data are for tissues from 5 mice/group.

The phyla Firmicutes, Bacteroidetes, and Tenericutes comprised 97% and 88% of the identified sequences in the cecal and colonic tissue of control C57BL/6J mice, respectively. The relative abundances of Firmicutes and Tenericutes were significantly reduced when C57BL/6J mice were fed diet with α-MG (*p <* 0.01). This change was associated with a significant increase in the relative abundance of Proteobacteria in the cecum and colon of these mice (*p <* 0.01), accounting for 86% and 82% of all sequences, respectively (Figure 3A). At the family level, dietary α-MG elicited a significant reduction in the relative abundance of *Erysipelotrichaceae*, *Lactobacillaceae*, *Rikenellaceae*, *Rumminococcaceae* and *Lachnospiraceae* (*p <* 0.01), while inducing a significant increase in the abundance of *Enterobacteriaceae* and *Enterococcaceae* in both cecum and colon (*p <* 0.01) (Figure 3B). At the genus level, *Allobaculum*, *Lactobacillus*, *Alistipes* and an unclassified genus within *Lachnospiraceae* were reduced in mice fed α-MG, while increases in *Enterobacter* and *Enterococcus* were observed.

The within-sample diversity was calculated using the Shannon index. Dietary α-MG elicited a significant reduction in bacterial diversity in the cecum and colon of C57BL/6J mice (*p <* 0.01) (Figure 3C,D). PCoA results indicated significantly different bacterial communities in the colon and cecum of these mice, as samples in the α-MG group clustered together and away from the control samples (Figure 3E,F). Accordingly, there were significant differences in the β-diversity between dietary groups (*p <* 0.01). 

#### 3.3.2. Balb/c Strain 

**Figure 4:**
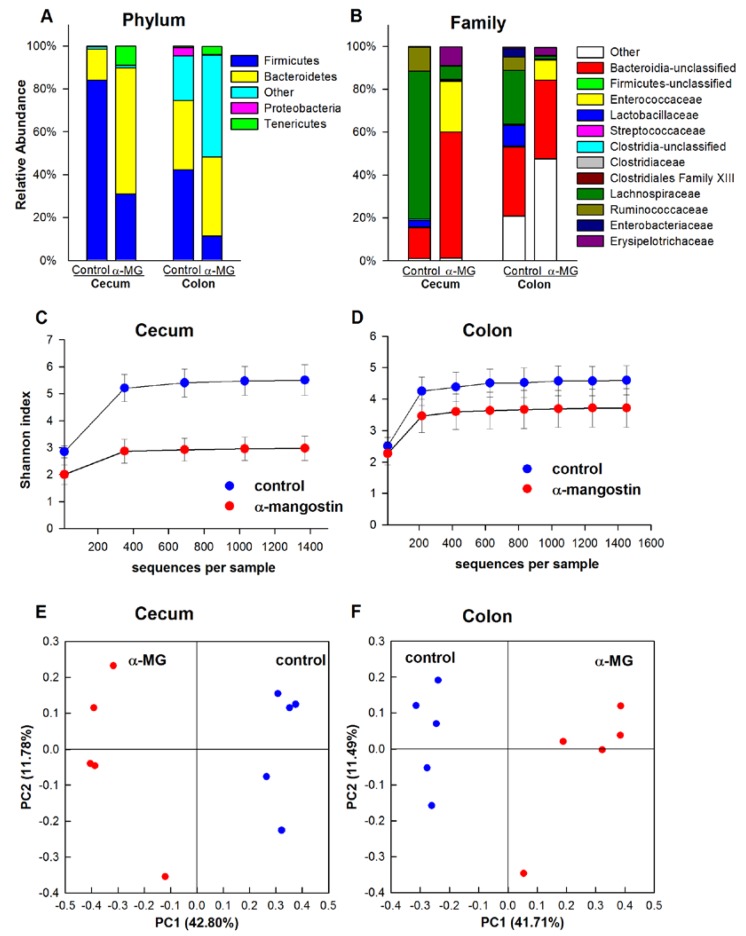
Dietary α-MG alters gut microbiota in Balb/c mice. Dietary α-mangostin (α-MG) changes the relative abundance of bacterial communities in the cecum and colon of Balb/c mice at the (**A**) phylum and (**B**) family levels. Significantly lower α-diversity in (**C**) cecum and (**D**) colon of mice fed α-MG. Panels E and F show principal coordinate analysis (PCoA) for cecum and colon, respectively. Samples in the α-MG group (red) are well separated from those in control group (blue). Data are for tissues from 5 mice/group.

Firmicutes and Bacteroidetes accounted for 84% and 14%, respectively, of all sequences in the cecum of control Balb/c mice. These proportions were significantly altered by dietary α-MG with Fimicutes and Bacteroidetes accounting for 59% and 31% of the community, respectively (*p <* 0.01), and the presence of significantly increased abundance of Tenericutes (*p <* 0.05). In the colon, Firmicutes significantly decreased in α-MG-fed animals (*p <* 0.01), and the relative abundance of Bacteroidetes remained unchanged. Greater relative abundance of Tenericutes and unclassified sequences at the phylum level also was observed in colon of mice fed α-MG, although these changes failed to achieve statistical significance (Figure 4A). The abundance of the Firmicutes family members *Lachnospiraceae* and *Ruminococcaceae* in the cecum was significantly reduced (*p <* 0.01). In addition to these changes, *Lactobacillaceae* was significantly decreased (*p <* 0.01) in the colon of Balb/c mice fed a diet containing α-MG. There also was a significantly greater abundance of an unclassified family within Bacteroidetes in the cecum of Balb/c mice fed α-MG (*p <* 0.01). The relative abundance of *Enterococcaceae* was greatly increased in both cecum and colon of mice fed diet with α-MG (*p <* 0.01) (Figure 4B). At the genus level, *Lactobacillus* and an unclassified genus within *Lachnospiraceae* were reduced in mice fed α-MG, whereas the relative abundance of *Enterococcus* was increased.

There was a significant reduction in the within-sample bacterial diversity in the cecum of Balb/c mice in the α-MG group (*p <* 0.01) (Figure 4C). Reduced α-diversity was also observed in the colon (*p* = 0.05) (Figure 4D). The bacterial communities in the colon and cecum of Balb/c mice analyzed by PCoA revealed marked separation of samples according to the dietary treatment group (Figure 4E,F). Significant differences in the β-diversity between dietary groups also were observed (*p <* 0.01). 

#### 3.3.3. C3H Strain

**Figure 5:**
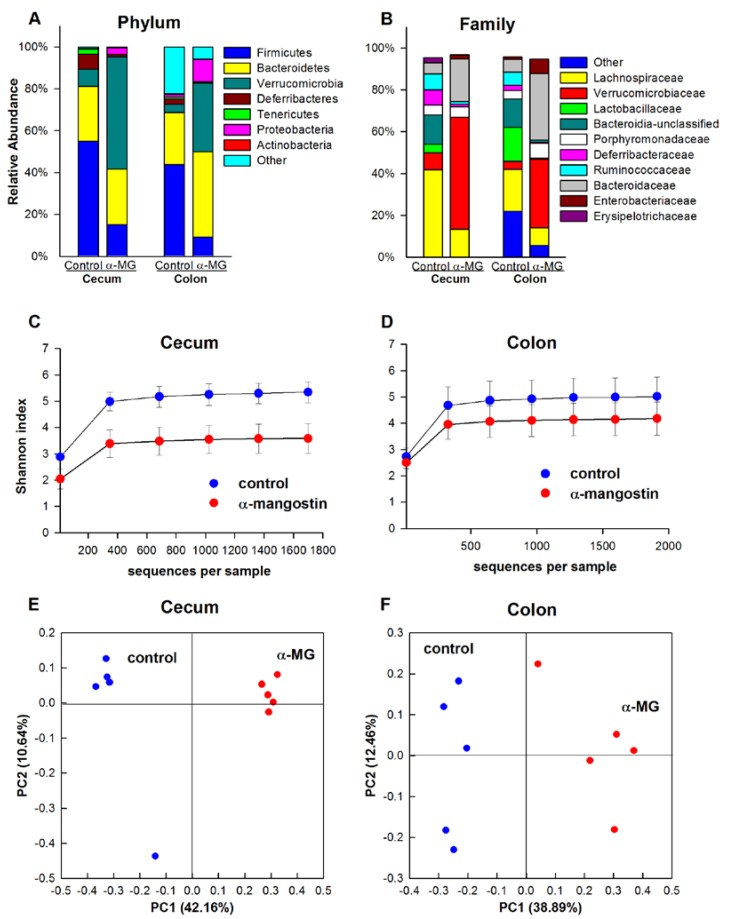
Dysbiosis is induced in colon and cecum of C3H mice fed diet with α-MG. Dietary α-mangostin (α-MG) changes the relative abundance of bacterial communities in the cecum and colon of C3H mice at the (**A**) phylum and (**B**) family levels. Mice fed α-MG had significantly lower α-diversity in (**C**) cecum but not in (**D**) colon. Panels E and F show principal coordinate analysis (PCoA) for cecum and colon, respectively. Samples in the α-MG group (red) cluster together and away from those in control group (blue). Data are for tissues from 5 mice/group.

Firmicutes and Bacteroidetes accounted for 55% and 26% of all sequences in the cecum of C3H control mice. The relative abundance of Firmicutes was significantly reduced by dietary α-MG to 15% (*p <* 0.05), whereas that of Bacteroidetes was not altered. There also was a significant increase in the abundances of Verrucomicrobia and Proteobacteria (*p <* 0.01). While Firmicutes was significantly decreased in the colon of C3H mice in the α-MG group (*p <* 0.01), the relative abundances of Verrucomicrobia, Bacteroidetes, and Proteobacteria significantly increased (*p <* 0.05) (Figure 5A). Significantly reduced cecal abundance of *Lachnospiraceae*, *Deferribacteraceae*, *Ruminococcaceae*, *Lactobacillaceae*, and an unclassified family within *Bacteroides* were observed in mice fed diet with α-MG (*p <* 0.01). In addition, *Lactobacillaceae* significantly decreased in the colon in response to dietary α-MG (*p <* 0.05) and the abundance of *Bacteroidaceae*, *Enterobacteriaceae*, and *Verrucomicrobiaceae* increased in both cecum and colon of C3H mice fed α-MG (*p <* 0.05). The abundance of *Lactobacillaceae*, *Ruminococcaceae*, and an unclassified family within *Bacteroidetes* in the colon of C3H mice fed α-MG also was decreased (Figure 5B). At the genus level, *Lactobacillus*, *Mucispirillum*, and an unclassified genus within *Lachnospiraceae* were reduced in mice fed α-MG, whereas there were increases in the abundance of *Bacteroides*, *Enterobacter*, and *Akkermansia*.

C3H mice fed α-MG had a significant reduction in the within-sample bacterial diversity in the cecum (*p <* 0.01), but not in the colon (Figure 5C,D). Results from the PCoA revealed significantly different bacterial communities in the colon and cecum of C3H mice fed diet with α-MG compared to those fed the control diet (Figure 5E,F). Significant differences in the β-diversity between dietary groups also were observed (*p <* 0.01). 

#### 3.3.4. Athymic FoxN1^nu^ Strain

**Figure 6:**
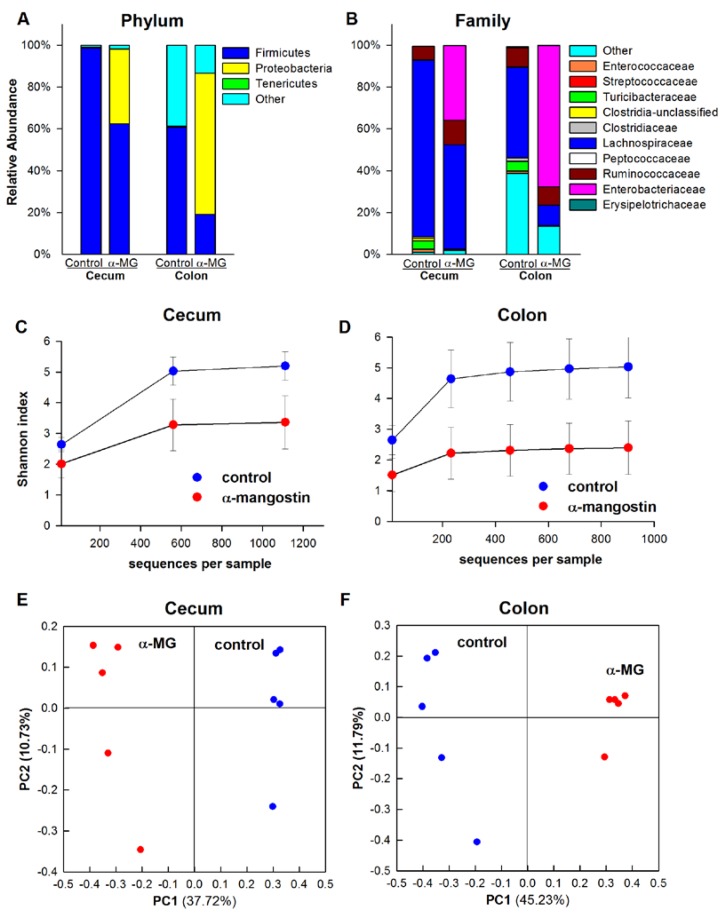
Altered composition in the microbiota of athymic FoxN1^nu^ mice fed diet containing α-MG. Dietary α-mangostin (α-MG) changes the relative abundance of bacterial communities in the cecum and colon of athymic FoxN1^nu^ mice at the (**A**) phylum and (**B**) family levels. Mice fed α-MG had significantly lower α-diversity) in (**C**) cecum and (**D**) colon compared to mice fed control diet. Panels E and F show principal coordinate analysis (PCoA) for cecum and colon, respectively. Samples in the α-MG group (red) cluster together and away from those in control group (blue). Data are for tissues collected from 5 mice/group.

Firmicutes accounted for 99% and 61% of all sequences in the cecum and colon, respectively, of athymic FoxN1^nu^ mice fed the standard AIN-93G diet. The relative abundance of Firmicutes in the cecum and colon of mice fed diet with α-MG was reduced to 62% and 19%, respectively (*p <* 0.01). These changes were associated with an increase in Proteobacteria in cecum and colon (*p <* 0.01) (Figure 6A). At the family level, *Lachnospiracea* and *Turicibacteriacea* were reduced in cecum and colon of mice fed α-MG (*p <* 0.01). Significant increases in the abundance of *Enterobacteriaceae* in the cecum and colon was also observed in this group (*p <* 0.01) (Figure 6B). The relative abundance of two genera, *Enterobacter* and *Anaerotruncus*, significantly increased in the colon and cecum of mice fed the diet containing α-MG.

The α-diversity (as measured by the Shannon index) was significantly reduced in the cecum and colon of mice fed α-MG (*p <* 0.01) (Figure 6C,D). These tissues also had significantly different microbial communities when compared to those from control animals, as seen from the PCoA analysis (Figure 6E,F). The β-diversity also was significantly different between dietary groups in both cecum and colon (*p <* 0.01).

## 4. Discussion 

Mangosteen is a fruit native to Southeast Asia where hot water extracts of pericarp have been used in traditional medicine to treat infected wounds, diarrhea and chronic ulcers [19]. *In vitro* and animal studies demonstrate that α-MG has anti-inflammatory [41,42,43], anti-cancer [44], and anti-microbial [20,45] activities. Supplements containing mangosteen are advertised by the nutraceutical industry as beneficial for gut heath and immunity, among other claimed health promoting properties. However, a recent study by our group indicated that dietary α-MG, the most abundant xanthone in mangosteen pericarp, exacerbated colonic injury and inflammation in colitic C57BL/6J mice, and increased fluid content of stools, infiltration of immune cells and the hyperproliferation of epithelial cells in the colon of non-colitic animals. The dietary xanthone also induced dysbiosis in the cecum and colon of otherwise healthy C57BL/6J mice [22]. 

The objective of the present study was to determine if the changes in the microbiota and colonic epithelium associated with ingestion of α-MG were specific to the C57BL/6J mouse strain or represented a more generic response to the xanthone among mice with diverse genetic backgrounds, each with unique microbiome composition. Host genetics affect both the response to chemical induction of colitis [46] and the composition of the gut microbiota [40]. The results of this study clearly show that feeding a diet containing 0.1% α-MG for 4 weeks to otherwise healthy C57BL/6J, C3H, Balb/c, and athymic FoxN1^nu^ strains of mice induced an increase in fluid content in stools in the absence of alterations in food intake or body weight. Moreover, chronic consumption of the diet with α-MG induced dysbiosis in the colon and cecum of these murine strains. These changes in the tissue-associated microbiota induced by α-MG resemble those reported in individuals with IBD [7,47,48]. Greater colonic epithelial cell proliferation and infiltration of T lymphocytes and macrophages were also observed in the colon of α-MG-fed mice. Our findings suggest that ingestion of mangosteen products may have unintended effects on health status, especially on individuals predisposed to or afflicted with inflammatory bowel disorders.

Inflammation is a component of many chronic diseases processes including cancer and cardiovascular disease [49]. Dietary polyphenols have been reported to suppress of pathology associated with excessive inflammation [50]. Various investigators have reported that α-MG also exerts anti-inflammatory activities in cellular and animal models [21]. Less attention has been given to the potential impact of these plant secondary metabolites such as α-MG and other xanthones on the microbiome [17]. As for other polyphenols, the majority of the studies addressing the anti-microbial activity of α-MG have used a single microorganism as the target rather than complex microbial communities such as those that exist in the large intestine. We are aware of a single report demonstrating the ability of α-MG to inhibit the growth of commensal bacteria [45], supporting the possibility that dietary α-MG can alter the complex microbial communities in the cecum and colon. Our results clearly demonstrate that chronic ingestion of α-MG induced marked alterations in the colonic and cecal microbiota of all four murine strains selected for the present investigation. The extent and type of these disruptions differed among strains but shared several common features. For instance, α-MG decreased the relative abundance of the phylum Firmicutes and its families *Lachnospiraceae*, *Ruminococcaceae*, and *Lactobacillaceae*. Also, the relative abundance of Proteobacteria and its *Enterobacteriaceae* family and the Firmicutes family *Enterococcaceae* increased in response to dietary α-MG. 

Firmicutes is the most abundant (64%) bacterial phylum in the human gut, while members from the Proteobacteria phylum are found in relatively lower abundance (8%) within the human gut microbiota [7]. This suggests that the alterations induced by α-MG may have important health implications. Reduced abundance of Firmicutes and increased abundance of Proteobacteria have been observed in individuals with IBD [47,48]. Reduced abundance of the Firmicutes family members *Rumminococcaea* and *Lachnospiraceae* is likely to affect the production of short chain fatty acids which are known to have anti-inflammatory activities and serve as fuel for colonic epithelial cells [51,52]. Similar to our results, the polyphenols quercetin and naringenin have been shown to inhibit the growth of *Lactobacillus* species in a dose dependent manner [53]. This bacterial group is predominant in the gut and it is linked to beneficial effects in the colon, such as the inhibition of growth of pathogenic bacterial species and the production of organic acids such as acetate and lactate that are utilized as fuel by colonic epithelial cells [54]. 

The increase in Proteobacteria in the colon and cecum of mice fed α-MG was primarily associated with an increase in *Enterobacteriaceae*. A similar increase in Proteobacteria has been reported in mice fed a diet containing polyphenols from black tea and red wine grapes, although such change was largely due to greater abundance of *Klebsiella sp*., a commensal opportunistic pathogen [55]. The mechanisms by which polyphenols influence the composition of non-pathogenic microbial communities in the gut remain unknown. C3H mice fed a diet containing α-MG also had increased abundance of Verrucomicrobia. Interestingly, this phylum is increased in the human gut after exposure to broad-spectrum antibiotics [56]. Dietary α-MG also increased the abundance of genera such as *Alistipes* and *Akkermansia*. Black tea and a red wine grape extract promoted the growth of *Akkermansia*, and the red wine grape extract also stimulated an increase in *Alistipes* [55]. *Akkermansia* participates in the degradation of mucin [57], and like *Alistipes,* metabolize polyphenols [55,58]. 

Because the absorption of most polyphenols is inefficient, these compounds transit into the colon in significant quantities [16] where they can exert effects on and be metabolized by the gut microbiota. The absorption of xanthones in humans is relatively poor [23] and their concentrations in plasma are much lower than those found in the gastrointestinal tract [24]. It is possible that xanthones and/or their metabolites modulate crosstalk among the colonic epithelium, the gut microbiota and the gut immune system. Because balanced interactions among epithelium, microbiota, and immune system are crucial for maintaining health, disruption of the balance (dysbiosis) may predispose to a diseased state. More research is needed on the role of dietary bioactive compounds and/or their metabolites in modulating the gut microbiome. Whether α-MG is metabolized by gut microbes remains unknown, but the increased abundance of some groups of bacteria in response to the ingestion of the xanthone suggests that these groups may preferentially be able to catabolize the xanthone or its products to support their growth. Elucidation of primary targets for the anti-bacterial activity of α-MG in the complex cecal and colonic environments also requires investigation, as does the reversibility of the changes in the microbiota following cessation of ingestion of α-MG. It is possible that the increased infiltration of pro-inflammatory immune cells in the colon may be a response of the host to the microbial changes. The gut microbiota, however, can also be altered in response to host mediated inflammation [9]. The sequence of changes induced by α-MG requires further investigation to determine if dysbiosis is a direct effect of the xanthone or the result of a pro-inflammatory immune response of the host to α-MG that in turn mediates changes in the microbiota. 

The present study was limited to examining the effect of a single dose (0.1% wt/wt) of α-MG. This dietary dose was selected for investigation because it was shown to decrease tumor growth in xenograft models of colon and prostate cancer [24,29], although it exacerbated murine colitis in our recent study [22]. This dose is achievable for individuals ingesting one to two capsules of pure α-MG dietary supplement, approximately seven capsules of a commercially available mangosteen dietary supplement containing 80 mg α-MG/capsule [29], or eight ounces (224 mL) of 100% mangosteen juice [23]. Studies are now needed to determine if the effects of dietary α-MG on the gut microbiota are dose dependent in mice and whether mangosteen-containing beverages and supplements also alter the microbiota and disease processes in humans.

## 5. Conclusions

Our results suggest that ingestion of α-MG may adversely impact the gut microbiota. These alterations resemble some of the changes present in the microbiota of individuals with IBD. The level of α-MG tested in our study can be achieved by ingesting available mangosteen-containing beverages and supplements. Thus, we suggest that consumption of pharmacological doses of xanthones in mangosteen-containing supplements may have a negative impact on the gut microbiota and their use should be considered with caution pending further information. 
